# The impact of condom use on the HIV epidemic

**DOI:** 10.12688/gatesopenres.13278.2

**Published:** 2022-02-11

**Authors:** John Stover, Yu Teng

**Affiliations:** 1 Center for Modeling and Analysis, Avenir Health, Glastonbury, CT, 06033, USA

**Keywords:** Condoms, HIV prevention, modeling

## Abstract

**Background: **Condom promotion and supply was one the earliest interventions to be mobilized to address the HIV pandemic. Condoms are inexpensive and provide protection against transmission of HIV and other sexually transmitted diseases (STIs) as well as against unintended pregnancy. As many as 16 billion condoms may be used annually in all low- and middle-income countries (LMIC). In recent years the focus of HIV programs as been on testing and treatment and new technologies such as PrEP. Rates of condom use have stopped increasing short of UNAIDS targets and funding from donors is declining.

**Methods: **We applied a mathematical HIV transmission model to 77 high HIV burden countries to estimate the number of HIV infections that would have occurred from 1990 to 2019 if condom use had remained at 1990 levels.

**Results: **The results suggest that current levels of HIV would be five times higher without condom use and that the scale-up in condoms use averted about 117 million HIV infections.

**Conclusions:** HIV programs should ensure that affordable condoms are consistently available and that the benefits of condom use are widely understood.

## Introduction

The distribution and promotion of condoms has been a part of efforts to prevent HIV transmission since the beginning of the HIV response. Early programs often focused on ABC (Abstinence, Be faithful, use Condoms). Condoms provide triple protection, against the transmission of HIV and other sexually transmitted infections as well protection against unintended pregnancy ^1^. Condom social marketing programs were the first HIV programs to reach national scale in many countries. The number of condoms distributed through social marketing programs increased from about 590 million annually in 1991 to 2.5 billion by 2012 before declining to about 1.7 billion in 2019 ^2^. Across 55 countries with a recent national household survey as part of the Demographic and Health Surveys (DHS) or AIDS Indicator Surveys (AIS) about 60 percent of men reported using a condom the last time they had sex with a non-marital, non-cohabiting partner and 65 percent report using a condom the last time they visited a sex worker ( Table 1).

**Table 1.** Reported rates of condom use at last sex with a higher risk partner and with a sex worker.

Country	Year and survey	Percentage reporting condom use at last higher risk sex	Percentage reporting condom use at last paid sex	Country	Year and survey	Percentage reporting condom use at last higher risk sex	Percentage reporting condom use at last paid sex
Albania	2017–18 DHS	58	65	Kenya	2014 DHS	76	74
Angola	2015–16 DHS	53	71	Kyrgyz Republic	2012 DHS	83	95
Armenia	2015–16 DHS	82	84	Lesotho	2014 DHS	77	90
Azerbaijan	2006 DHS	35	53	Liberia	2013 DHS	42	61
Benin	2017–18 DHS	36	44	Madagascar	2008–09 DHS	13	13
Bolivia	2008 DHS	50	89	Malawi	2015–16 DHS	73	75
Burkina Faso	2010 DHS	74	33	Mali	2018 DHS	39	70
Burundi	2016–17 DHS	51	55	Moldova	2005 DHS	54	
Cambodia	2014 DHS	74	82	Mozambique	2015 AIS	47	31
Cameroon	2018 DHS	63	83	Myanmar	2015–16 DHS	77	77
Chad	2014–15 DHS	42	50	Namibia	2013 DHS	80	67
Colombia	2015 DHS	71	85	Nepal	2016 DHS	68	93
Comoros	2012 DHS	60	65	Niger	2012 DHS	64	
Congo	2011–12 DHS	58	75	Nigeria	2018 DHS	65	74
Congo Democratic Republic	2013–14 DHS	31	34	Papua New Guinea	2016–18 DHS	33	48
Cote d'Ivoire	2011–12 DHS	63	63	Philippines	2003 DHS	24	36
Dominican Republic	2013 DHS	71	80	Rwanda	2014–15 DHS	66	65
Eswatini	2006–07 DHS	67		Sao Tome and Principe	2008–09 DHS	61	76
Ethiopia	2016 DHS	51	81	Senegal	2019 DHS	72	
Gabon	2012 DHS	75	83	Sierra Leone	2019 DHS	23	57
Gambia	2013 DHS	67	69	South Africa	2016 DHS	73	83
Ghana	2014 DHS	39	44	Tanzania	2011–12 AIS	60	
Guatemala	2014–15 DHS	68	80	Timor-Leste	2016 DHS	34	40
Guinea	2018 DHS	50	72	Togo	2013–14 DHS	61	62
Guyana	2009 DHS	72	82	Uganda	2016 DHS	62	73
Haiti	2016–17 DHS	63	90	Ukraine	2007 DHS	62	84
Honduras	2011–12 DHS	61	32	Vietnam	2005 AIS	73	
India	2015–16 DHS	41	48	Zambia	2018 DHS	54	56
Indonesia	2012 DHS		34	Zimbabwe	2015 DHS	82	90

Note: ‘Higher risk sex’ refers to sex with a non-marital, non-cohabiting partner. Blank cells represent missing data. Data accessed on May 24, 2017 through the StatCompiler tool available from the Demographic and Health Survey project at http://www.statcompiler.com/en/.

In all low- and middle-income countries about 16 billion condoms are used annually with about 7.5 billion used primarily for HIV prevention ^1^. Since these figures are based on self-reports of condom use, they may over-state actual use. However, it is clear that large numbers of condoms have been procured and/or distributed with the intention of helping users prevent HIV transmission.

Studies have shown condoms to be highly effective against HIV ^3^, other sexually transmitted infections ^4^ and unintended pregnancy ^5^. Consistent use is required to maximize an individual’s protection. However, even inconsistent use will provide some benefit that can be large at a population-level ^6^.

Across all DHS surveys about three-fifths of people report relying on the public sector for their condom supply. Social marketing programs provide nearly 2 billion condoms each year ( https://www.dktinternational.org/contraceptive-social-marketing-statistics/), about Thus, international donor and national government funding for condom purchase, distribution and promotion plays a large role in supporting the widespread use of condoms.

The purpose of this paper is to investigate the global impact of condoms on the HIV epidemic through both retrospective and prospective analyses.

## Methods

We used a publicly available mathematical simulation model, the Goals model ^7^, to examine the impact of past and future condom use on the AIDS epidemic in 77 high burden countries. We used version 6.06 of the Goals model, which is available for free download at https://www.avenirhealth.org/software-spectrum.php. The source code for the calculations is available as *Extended data* ^8^. This is the same model that was used to estimate epidemiological impact for the new UNAIDS Global HIV Strategy ^9^.

Goals is a simulation model that calculates HIV transmission among different population risk groups (monogamous heterosexual couples, those with multiple heterosexual partners, female sex workers and clients, men who have sex with men (MSM), and people who inject drugs (PWID)) on the basis of their behaviors (number of partners, contacts per partner, condom use, age at first sex, needle sharing) and characteristics that influence transmission (presence of other sexually transmitted infections, stage of infection, male circumcision, and use of antiretroviral therapy (ART) and pre-exposure prophylaxis (PrEP)). The model uses data on behaviors drawn from national surveys, such as DHS, and program data on the coverage of ART and programs to prevent mother-to-child transmission, PMTCT, from UNAIDS’ HIV database. The model is fit to official estimates of HIV prevalence trends for each county, also available from UNAIDS.

HIV transmission is calculated as a function of epidemiological factors and the behavioral factors listed above. For uninfected people in each risk group, the probability of becoming infected in a year is given by the following equation:

P _s,r,t_ =   {1-[Prev _s’,r,t_ × (1-r _s_ × S _s,r,t_ × STI _s,r,t_ × MC _t_ × C _r,t_ × PrEP _s,r,t_ × ART _s,r,t_) ^a^ + (1-Prev _s’,r,t_)] ^n^}

Where:

P _s,r,t_         = Annual probability of becoming infected for a person of sex *s* in risk group *r* at time *t*

Prev _s’,r,t_   = HIV prevalence in the partner population in risk group *r* at time *t*

r _s_             = probability of transmission per sex act by type of act (heterosexual, homosexual)

S _s,r,t_        = multiplier based on the stage of infection (primary stage, chronic stage or late stage)

MC _r,t_      = multiplier based on male circumcision status

STI _r,t_      = multiplier based on STI prevalence

C _r,t_         = multiplier based on condom use

PrEP _r,s,t_  = multiplier based on the use of PrEP

ART _s,t_    = multiplier based on ART use

a _r,t_          = number of acts per partner per year in risk group *g* at time *t*

n _r,t_          = number of partners per year in risk group *g* at time *t*

The multipliers on the probability of infection per act (MC, C, PrEP and ART) are based on the probability of circumcision, condom, PrEP or ART use and the effectiveness of each in preventing the transmission of HIV. Effectiveness rates used in this analysis are 0.6 for male circumcision ^10– 12^, 0.8 for condoms ^3^, 0.8 for PrEP ^13– 16^ and 0.95 for ART ^17^. The probability of infection per act and the STI and stage of infection multipliers are selected from within published ranges to best fit the epidemic in each country. Ranges are 0.0008 – 0.0016 for the probability of infection per act ^18, 19^, 2–11 for STIs ^20, 21^, 0.8–44 for primary stage infection ^22– 24^ and 4–12 for symptomatic stage infection ^22^. The number of contacts per partner and the number of partners per year are exponents in the equation to convert the risk per act into a cumulative risk of infection across all acts and all partners. Condom coverage represents the percentage of sexual acts that involve condom use. Since the model does not track individuals separately, it does not distinguish between consistent and inconsistent use. Each condom used has the effect of reducing the probability of transmission for that act. The cumulative impact across all acts is the net effect of condom use ^7^.

We applied the Goals model to 77 countries that together account for 94% of new infections globally in 2019 ( https://aidsinfo.unaids.org/) and then scaled-up the result to correspond to the global epidemic. The full list of countries included is in *Underlying data* ^8^. The model is implemented for each individual country by using country-specific data for demographic indicators (base year population, fertility, mortality, and migration) ( https://population.un.org/wpp/), behavioral indicators (number and type of partners, condom use) from national household surveys ( https://www.statcompiler.com/en/), and HIV program data (number of people on ART and number of women receiving prophylaxis to prevent mother-to-child transmission (PMTCT) and number of male circumcisions) ( https://aidsinfo.unaids.org/). The model is fit to data on prevalence from surveys, surveillance, and routine testing by varying the epidemiological parameters within published ranges. The ranges used for the epidemiological parameters and the fitted values by country are provided in the underlying data.Historical trends in condom use by population group were estimated from self-reported condom use in DHS. Reported condom use in commercial sex was used for sex worker contact, reported use among those engaging in higher-risk sex was used for those with multiple partners and reported condom use for contraception was used for those with one partner. Information on the size of key populations is from the UNAIDS Key Population Atlas ( https://kpatlas.unaids.org/).

Once the model was fit to each country’s actual epidemic we conducted three analyses: (1) a retrospective analysis that estimates the number of additional HIV infections that would have happened if condom use rates stayed constant from 1990 to 2019, (2) a prospective analysis that compares the number of new HIV infections expected to occur between 2020 and 2030 if condom use rates remain at 2019 levels or increase to reach UNAIDS targets of 95% of casual and sex work contacts protected by condom use by 2025, and (3) a prospective analysis that compares constant condom use rates from 2019 to 2030 with a future where all key HIV interventions increase to UNAIDS targets by 2030 ^25^ for key populations (sex workers, MSM, PWID, transgender people and prisoners), adolescent girls and young women, adolescent boys and young men, adults aged 25+, HIV-positive pregnant women and people living with HIV. Comprehensive services are targeted to the appropriate populations and include testing, treatment, condoms provision, needle and syringe exchange, opioid substitution therapy, PrEP, PEP comprehensive sexuality education, economic empowerment, voluntary medical male circumcision and prevention of mother-to-child transmission. These scenarios are illustrated in Table 2.

**Table 2.** Scenario descriptions.

Scenario	Condom coverage	Coverage of other prevention interventions
Retrospective: 1990–2019		
- Counterfactual	Constant at 1990 levels	Actual
- Actual	Actual	Actual
Prospective: 2020–2030		
- Counterfactual	Constant at 2019 levels	Constant at 2019 levels
- Condom scale-up	95% of casual and sex work contacts protected by condoms by 2025	Constant at 2019 levels
- UNAIDS targets	95% of casual and sex work contacts protected by condoms by 2025	Scale up to all UNAIDS targets by 2025

We tested the sensitivity of the model results to the assumed effective of condoms in averting HIV infection by also running simulations with the effectiveness of condoms set to the low end of the 95% confidence interval (0.50) and with the high end (0.94).

## Results

**Figure 1.**
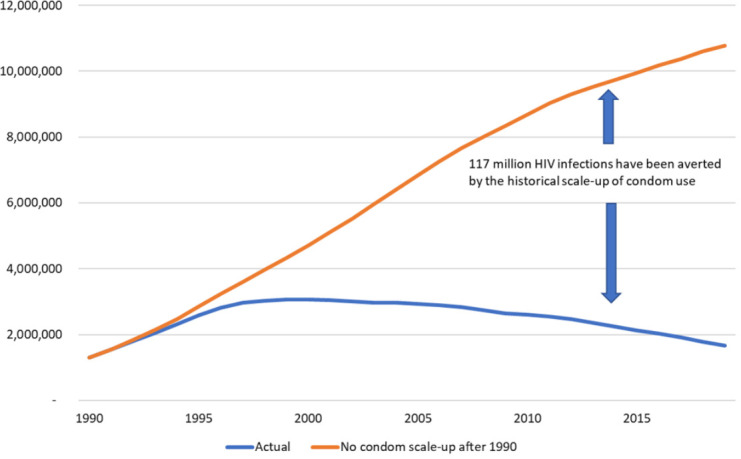
Number of new HIV infections with and without historical scale-up of condom use.

According to UNAIDS estimates, the annual number of new HIV infections worldwide increased to a peak of about 2.8 million around 1998 and then declined to 1.7 (1.2 – 2.2) million by 2019 ^26^. Model simulations with no increase in condom use rates after 1990 project that the annual number of new HIV infections would have increased to nearly 11 million by 2019 ( Figure 1).

The difference between the lines represents 117 million infections averted from 1990–2019 due to increased condom use. Without the condom scale-up the cumulative number of new infections would have been 160 percent larger. About 45% of the estimated infections averted are in sub-Saharan Africa, 37% in Asia and the Pacific, 10% in Latin America and the Caribbean and 4% each in the Eastern Europe and Central Asia region and the Western and Central Europe and North America region. Impact for each of the modeled countries is shown in the *Underlying data* ^8^. The largest absolute impacts, in terms of infections averted, are seen in the countries with the largest populations or highest prevalence (South Africa, India, China, Kenya and Tanzania) while the highest relative impact occurs in countries with low burden currently where condom use helped to avert a larger epidemic (Guatemala, China, United Kingdom, Italy, Mongolia and Bangladesh).

The sensitivity analysis of condom effectiveness indicates that the estimate of 117 million infections averted could be as low as 70 million or as high as 130 million.

We do not know how many condoms were used globally between 1990 and 2019 but if we assume that condom use was very low in 1990 and scaled up to near today’s rates by 2010 and remained approximately constant from 2010 to 2019, then total condom consumption for HIV prevention would have been around 160 billion for that period. This implies a global average of about 1300 condoms per infection averted. At an average cost per condom distributed of about $0.18 ^27^ the cost per infection averted by condoms during 1990–2019 is about $230.170

**Figure 2.**
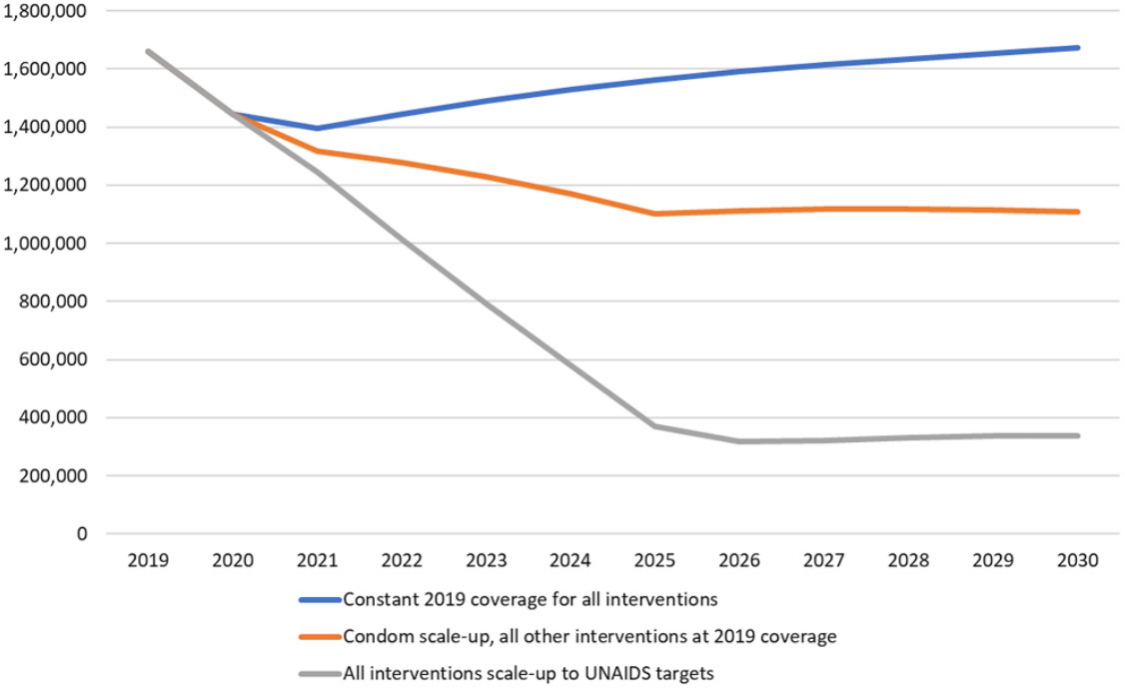
Number of new HIV infections in the future under three scenarios.

Figure 2 shows the two projections from 2019 to 2030. If condom use rates remained at their 2019 levels and all other interventions also had constant coverage, then the annual number of new HIV infections would rise slowly due to constant incidence and a growing population. If condom use rates scaled-up everywhere to the UNAIDS target of 95% of all risky sex acts and all other prevention interventions remained at 2019 coverage levels, then the number of new infections would decline to 1.1 million 2030. The difference between these two lines indicates that condom scale-up would avert about 3.6 million HIV infections over that period, about 20% of those that would occur without condom scale-up. Figure 2 also shows that the rapid scale-up of condom use could produce about one-third the impact as the full UNAIDS strategy, which scales up all the intervention mentioned above to UNAIDS targets.

## Discussion

Condom use has increased dramatically since the beginning of the HIV epidemic. Today, approximately 16 billion condoms are used annually to prevent infections and unintended pregnancies. Condom use has impacted the HIV epidemic and avoided a much worse HIV epidemic than has actually evolved. Condoms can play a key role in future efforts, such as the Fast-Track initiative to end AIDS as a public health threat by 2030 ^28^.

The number of HIV new infections under the retrospective counterfactual scenario of no increase in condom use after 1990, which reaches 11million by 2019, is quite high compared to the actual level of about 1.7 million. But this just illustrates the benefits of early intervention. Early increases in condom use among key populations, in particular sex workers and their clients, as well as with non-regular partners has slowed early transmission and helped to avert a much larger epidemic in the general population.

There are several limitations to this analysis. We rely on self-reports of condom use in national surveys that may over-state actual use. The effectiveness of condoms depends on correct and consistent use but measures of these factors are not well developed. Our modeling estimates the impact of condom use in aggregate population groups but does not model individual behavior. Using these data our models can replicate historical epidemic trends in the countries modeled but that does not ensure that they are correct. Findings of this analysis are, however, broadly consistent with other mathematical modelling analyses of the impact of condom use ^29, 30^. Estimates of the size of key populations in each country are based on small sample surveys which may not be representative of the entire country. Estimates of the number of acts per partner are based on small studies or potentially unreliable self-reports. To some extent, these limitations are addressed by fitting the model to historical data on prevalence. While the fitting does not guarantee that all the inputs are correct, it does ensure that the set of inputs is sufficient to replicate the historical epidemic. In spite of above-mentioned limitations, the case for the importance of condoms as an ongoing component of HIV programming is compelling.

Previous modeling studies have shown the impact of historical condom scale-up in specific populations in specific-countries including sex workers in Benin ^31^ and MSM in Beijing, China ^32^. Other studies have modeled the potential impact of programs to scale-up condom use, including adolescents in the United States ^33^ and hypothetical but representative settings ^34^. All found significant impacts of condom use, but none examined the global impact. Condoms are a good investment. The total cost to prevent one new HIV infection with condoms is small compared to life-time costs of treatment meaning that condom investments now will save future expenditures on treatment. Since many people rely on free or subsidized condoms, it is crucial to ensure adequate funding for condom programs, including demand creation activities and frequent behavioral data collection.

While condoms are not a magic bullet that alone can control the HIV epidemic, they remain a critical part of the prevention response. Scale-up of condoms use is a necessary component to reach the UNAIDS global targets ^9^ and any reduction in support for condoms would seriously affect the changes of achieving those targets. Unfortunately, support for condom social marketing programs has been decreasing in recent years ^35^. International and domestic financing should continue to support general population condom programs even as new technologies are introduced that are targeted to the highest risk populations. Condom programs remain among the most cost-effective interventions in the response and provide other health benefits including prevention of other sexually transmitted infections and protection against unwanted pregnancies ^1^. Past experience has shown that we do know how to promote and distribute condoms and that many people will use them if they are available. Recent declines in condom investments especially around demand creation implies that the younger generation have not been exposed to relevant condom promotion and condom use skills, a worrisome trend given the relative size of young populations in low- and middle-income countries.

## Data availability

### Underlying data

Zenodo: JGStover/Data-for-condom-impact-paper-on-Gates-Open-Research: Impact of condoms. https://doi.org/10.5281/zenodo.4898086 ^8^.

This project contains the following underlying data:

- Appendix Table 1.csv (number of new HIV infections by country from 1990–2019 according to actual trends or a counterfactual scenario in which rates of condom use remain at 1990 levels)

Zenodo: JGStover/Data-for-condom-impact-paper-on-Gates-Open-Research: Impact of condoms. https://doi.org/10.5281/zenodo.4898086 ^8^.

This project contains the following extended data:

- Parameter ranges used for model fitting.docx (the ranges for key epidemiological factors used in model fitting)
- Fitted parameter values by county.docx (final fitted values for key epidemiological parameters for each country)
- Calculation code (the Delphi code for the simulation calculations in the Goals in .PAS format)

Data are available under the terms of the Creative Commons Zero "No rights reserved" data waiver (CC0 1.0 Public domain dedication).
