# Supplementary material for: The impact of condom use on the HIV epidemic
Source: Gates Open Res. 2022 Feb 11;5:91. Originally published 2021 Jun 9. [Version 2] doi: 10.12688/gatesopenres.13278.2 (PMC8933340; doi:10.12688/gatesopenres.13278.2)
Supplement: Peer review history

# Peer review: Reviewer response for version 2

**DOI:** 10.21956/gatesopenres.14833.r31760

**Published:** 2022-03-18

**Authors:** Akira Shibanuma

**Affiliations:** 1 Department of Community and Global Health, Graduate School of Medicine, The University of Tokyo, Tokyo, Japan

Thank you very much for addressing the comments made in the previous round. The following is a minor comment related to the comment made in the previous round.

**Response**: We do provide a citation for the full model details when we say ‘Complete model details are available elsewhere [24].’ That reference provides equations and data sources. This is the same model used in the PLoS Medicine paper. We have added a citation to the PloS Medicine paper to make that clear. We have also added some clarification to the use of the number of acts per partner and the number of partners as exponents in the equation.

 Regarding the response to the reviewer above, I could not find the description (‘Complete model details are available elsewhere [24].’) in the version 1 of the manuscript. I understand that what the authors used in this manuscript is the Goals RSM (Risk-Structured Model) used in the Plos Medicine paper by the authors (Ref number 9 in the current version of the manuscript), and that the model detail was described in the web appendix of the author's different paper (Ref number 7). If this understanding is correct, the authors might want to clarify it in the manuscript for readers' better understanding of the model.

Is the work clearly and accurately presented and does it cite the current literature?

No

If applicable, is the statistical analysis and its interpretation appropriate?

Partly

Are all the source data underlying the results available to ensure full reproducibility?

No

Is the study design appropriate and is the work technically sound?

Yes

Are the conclusions drawn adequately supported by the results?

Partly

Are sufficient details of methods and analysis provided to allow replication by others?

No

Reviewer Expertise:

community health; health promotion; maternal, newborn, and child health; immigration and health; prevention and control of infectious diseases

I confirm that I have read this submission and believe that I have an appropriate level of expertise to confirm that it is of an acceptable scientific standard.

# Peer review: Reviewer response for version 2

**DOI:** 10.21956/gatesopenres.14833.r31759

**Published:** 2022-02-25

**Authors:** Innocent Modisaotsile, Willis Odek

**Affiliations:** 1 United Nation Population Fund, UNFPA, New York, NY, USA; 2 UNFPA, New York, NY, USA

Thanks a lot for sharing with us the latest version. We have reviewed it and we think our observations were adequately addressed. We therefore approve the paper without any reservations.

Is the work clearly and accurately presented and does it cite the current literature?

Yes

If applicable, is the statistical analysis and its interpretation appropriate?

Yes

Are all the source data underlying the results available to ensure full reproducibility?

Yes

Is the study design appropriate and is the work technically sound?

Yes

Are the conclusions drawn adequately supported by the results?

Yes

Are sufficient details of methods and analysis provided to allow replication by others?

Yes

Reviewer Expertise:

NA

We confirm that we have read this submission and believe that we have an appropriate level of expertise to confirm that it is of an acceptable scientific standard.

# Peer review: Reviewer response for version 1

**DOI:** 10.21956/gatesopenres.14517.r31288

**Published:** 2021-11-05

**Authors:** Akira Shibanuma

**Affiliations:** 1 Department of Community and Global Health, Graduate School of Medicine, The University of Tokyo, Tokyo, Japan

This study developed a mathematical model for the incidence of HIV infection in 77 high HIV burden countries to estimate the difference in the incidence between the cases of the actual and hypothetical condom coverage among risk populations of HIV infection. This prevalence highlights the importance of promoting condom use among these populations. I hope the following points would help the authors update the manuscript.

  

1. Model: This manuscript does not describe the model in detail, unlike a modelling paper published by the authors (e.g., the PloS Medicine paper ^1^). The model for the incidence in the present study seems to differ substantially from the one in the PloS Medicine paper (Function (2) in S2 Text) (of course, the purpose of the modelling differed, too). Note that readers in this journal are not necessarily familiar with modelling studies. For example, readers may want to know different roles of Prev_s’,r,t × (1-r_s × S_s,r,t × STI_s,r,t × MC_t × C_r,t × PrEP_s,r,t × ART_s,r,t)^a and (1 - Prev_s’,r,t), reasons of using exponential functions with regard to the number of acts per partner and the number of partners. Although there is no citation for the model, the authors may want to add references if the model in the current study was built based on previous works.
2. Values to be input in the model: The authors may want to describe how values of several key variables were obtained, such as the coverage of condom use among each of risk populations in 1990 and onward in each country, the estimated number of these key populations in the past, present, and future years. The authors may want to describe assumptions in the estimated values, if any, in the Methods section and in the limitations in the Discussion section. In addition, values that were input in the model may need to be attached so that readers can verify the validity of the modelling.
3. Sensitivity analysis: For future estimations, the authors may need to consider sensitivity analysis for different parameters and the past and future values of key variables. For example, it may not be realistic to have fixed values for the number of acts per partner and the number of partners among different key populations in the past or future years. In some countries, it may be difficult to obtain reliable sources for the current statistics for these numbers.
4. Discussion: The discussion section does not contain the interpretations of findings, comparisons of findings in this study with ones in previous studies, and implications for the global targets related to HIV/AIDS.

Is the work clearly and accurately presented and does it cite the current literature?

No

If applicable, is the statistical analysis and its interpretation appropriate?

Partly

Are all the source data underlying the results available to ensure full reproducibility?

No

Is the study design appropriate and is the work technically sound?

Yes

Are the conclusions drawn adequately supported by the results?

Partly

Are sufficient details of methods and analysis provided to allow replication by others?

No

Reviewer Expertise:

community health; health promotion; maternal, newborn, and child health; immigration and health; prevention and control of infectious diseases

I confirm that I have read this submission and believe that I have an appropriate level of expertise to confirm that it is of an acceptable scientific standard, however I have significant reservations, as outlined above.

## References

[1] : Modeling the epidemiological impact of the UNAIDS 2025 targets to end AIDS as a public health threat by 2030. *PLoS Med* .18(10) : 10.1371/journal.pmed.1003831 e1003831 10.1371/journal.pmed.1003831 34662333PMC8559943

# Response

**Published:** 2022-02-04

**Authors:** John Stover

**Affiliations:** Avenir Health, USA

Reviewer #2

**Akira Shibanuma**, Department of Community and Global Health, Graduate School of Medicine, The University of Tokyo, Tokyo, Japan 

 This study developed a mathematical model for the incidence of HIV infection in 77 high HIV burden countries to estimate the difference in the incidence between the cases of the actual and hypothetical condom coverage among risk populations of HIV infection. This prevalence highlights the importance of promoting condom use among these populations. I hope the following points would help the authors update the manuscript.

  

- Model: This manuscript does not describe the model in detail, unlike a modelling paper published by the authors (e.g., the PloS Medicine paper ^1^ ). The model for the incidence in the present study seems to differ substantially from the one in the PloS Medicine paper (Function (2) in S2 Text) (of course, the purpose of the modelling differed, too). Note that readers in this journal are not necessarily familiar with modelling studies. For example, readers may want to know different roles of Prev_s’,r,t × (1-r_s × S_s,r,t × STI_s,r,t × MC_t × C_r,t × PrEP_s,r,t × ART_s,r,t)^a and (1 - Prev_s’,r,t), reasons of using exponential functions with regard to the number of acts per partner and the number of partners. Although there is no citation for the model, the authors may want to add references if the model in the current study was built based on previous works.

**Response**: We do provide a citation for the full model details when we say ‘Complete model details are available elsewhere [24].’ That reference provides equations and data sources. This is the same model used in the PLoS Medicine paper. We have added a citation to the PloS Medicine paper to make that clear. We have also added some clarification to the use of the number of acts per partner and the number of partners as exponents in the equation.

  

- Values to be input in the model: The authors may want to describe how values of several key variables were obtained, such as the coverage of condom use among each of risk populations in 1990 and onward in each country, the estimated number of these key populations in the past, present, and future years. The authors may want to describe assumptions in the estimated values, if any, in the Methods section and in the limitations in the Discussion section. In addition, values that were input in the model may need to be attached so that readers can verify the validity of the modelling.

**Response**: We have added a description of the sources of information on historical condom use and the estimation of key population sizes. Table 1 shows the reported condom use rates by population group and country.

  

- Sensitivity analysis: For future estimations, the authors may need to consider sensitivity analysis for different parameters and the past and future values of key variables. For example, it may not be realistic to have fixed values for the number of acts per partner and the number of partners among different key populations in the past or future years. In some countries, it may be difficult to obtain reliable sources for the current statistics for these numbers.

**Response**: Yes, we agree that these inputs are not well known and have added some text in the limitations paragraph to acknowledge this.

  

- Discussion: The discussion section does not contain the interpretations of findings, comparisons of findings in this study with ones in previous studies, and implications for the global targets related to HIV/AIDS.

**Response**: We have added comparisons to four other studies and expanded the discussion to include implications for the global targets.

# Peer review: Reviewer response for version 1

**DOI:** 10.21956/gatesopenres.14517.r30763

**Published:** 2021-07-01

**Authors:** Innocent Modisaotsile, Willis Odek

**Affiliations:** 1 United Nation Population Fund, UNFPA, New York, NY, USA; 2 UNFPA, New York, NY, USA

This study presents a retrospective (since 1990) and prospective (up to 2030) analysis of the role of condoms in averting new HIV infections using the Goal Model in 77 countries. The model parameters are clearly spelled out and justified. The analysis uses data collected through representative general population surveys. While the analysis focuses on the role of condoms in averting new HIV infections, it also models the effect of other HIV prevention interventions on new HIV infections. We were concerned upfront about the accuracy of condom use estimates from general population surveys, but this limitation has been duly acknowledged by the authors and reflected in the interpretation of results with a caveat that the analysis does not measure consistent condom use, which is a behavioral factor.

 It would be helpful to clarify or elaborate further on the following:

1. The rationale for setting the baseline analysis period to 1990, especially given the emergence of new HIV prevention tools, including ART, and their roll out to most affected countries only towards the year 2000.
2. The formula for estimating the probability of becoming infected in a year includes a parameter on HIV prevalence of the opposite sex. Considering that in many regions, gay men and men who have sex with men contribute significantly to new infections (64% in West and central Europe, 44% in Asia and the pacific as well as Latin America – source: 'UNAIDS 2021, Global Commitment, Local Action - After 40 years of AIDS, charting a course to end the pandemic' (link to source available here) - how did we address this parameter?
3. The paper also indicates that private sector contributes 60% of condoms in at least 55 countries based on DHS. While this might be a global average, for other regions such as sub-Saharan Africa, the major contributor is the public sector, with the private sector contributing less than 20%. It might be worth noting the exceptions, especially given the importance of free condoms in the African continent..

Given its estimation of some 117 million new HIV infections averted since 1990 due to scale up of condom use, the paper should include a strong programmatic recommendation for effective integration of condom programming with other HIV prevention interventions, including sexual and reproductive health and rights.   

Is the work clearly and accurately presented and does it cite the current literature?

Yes

If applicable, is the statistical analysis and its interpretation appropriate?

Yes

Are all the source data underlying the results available to ensure full reproducibility?

Yes

Is the study design appropriate and is the work technically sound?

Yes

Are the conclusions drawn adequately supported by the results?

Yes

Are sufficient details of methods and analysis provided to allow replication by others?

Yes

Reviewer Expertise:

NA

We confirm that we have read this submission and believe that we have an appropriate level of expertise to confirm that it is of an acceptable scientific standard, however we have significant reservations, as outlined above.

# Response

**Published:** 2022-02-04

**Authors:** John Stover

**Affiliations:** Avenir Health, USA

Reviewer # 1

**Innocent Modisaotsile**, United Nation Population Fund, UNFPA, New York, NY, USA

**Willis Odek**, UNFPA, New York, NY, USA

 This study presents a retrospective (since 1990) and prospective (up to 2030) analysis of the role of condoms in averting new HIV infections using the Goal Model in 77 countries. The model parameters are clearly spelled out and justified. The analysis uses data collected through representative general population surveys. While the analysis focuses on the role of condoms in averting new HIV infections, it also models the effect of other HIV prevention interventions on new HIV infections. We were concerned upfront about the accuracy of condom use estimates from general population surveys, but this limitation has been duly acknowledged by the authors and reflected in the interpretation of results with a caveat that the analysis does not measure consistent condom use, which is a behavioral factor.

 It would be helpful to clarify or elaborate further on the following:

- The rationale for setting the baseline analysis period to 1990, especially given the emergence of new HIV prevention tools, including ART, and their roll out to most affected countries only towards the year 2000.

**Response**: While the scale-up of key programs such as ART, PMTCT and VMMC only took place after 2000, increases in condom use started much earlier. In the 1990s programs focused on ABC (Abstinence, Be Faithful and Condoms). We wanted to capture the full benefits of increases in condom use by starting the analysis in 1990.

  

- The formula for estimating the probability of becoming infected in a year includes a parameter on HIV prevalence of the opposite sex. Considering that in many regions, gay men and men who have sex with men contribute significantly to new infections (64% in West and central Europe, 44% in Asia and the pacific as well as Latin America – source: 'UNAIDS 2021, Global Commitment, Local Action - After 40 years of AIDS, charting a course to end the pandemic' (link to source available here) - how did we address this parameter?

**Response**: Thank you for catching that. The formula actually uses prevalence in the partner population whether the partner is the opposite or same sex. We have revised the variable description to show that.

  

- The paper also indicates that private sector contributes 60% of condoms in at least 55 countries based on DHS. While this might be a global average, for other regions such as sub-Saharan Africa, the major contributor is the public sector, with the private sector contributing less than 20%. It might be worth noting the exceptions, especially given the importance of free condoms in the African continent.

**Response**: We have updated the text to include the latest data from DHS and social marketing which indicate that 60-70% of those using condoms for contraception get them from public sources and social marketing accounts for nearly 2 billion condoms each year.

- Given its estimation of some 117 million new HIV infections averted since 1990 due to scale up of condom use, the paper should include a strong programmatic recommendation for effective integration of condom programming with other HIV prevention interventions, including sexual and reproductive health and rights.  

**Response**: We feel that we do make a strong recommendation for continued support for condom programming in the last paragraph of the discussion.
